# The hazards of death by smoking in middle-aged women

**DOI:** 10.1007/s10654-013-9851-6

**Published:** 2013-09-29

**Authors:** Inger T. Gram, Sven Sandin, Tonje Braaten, Eiliv Lund, Elisabete Weiderpass

**Affiliations:** 1 Department of Community Medicine, Faculty of Health Sciences, University of Tromsø, The Arctic University of Norway, Tromsö, Norway; 2 Norwegian Centre for Integrated Care and Telemedicine, University Hospital of North Norway, Tromsö, Norway; 3 Department of Medical Epidemiology and Biostatistics, Karolinska Institutet, Stockholm, Sweden; 4 Department of Genetic Epidemiology, Samfundet Folkhälsan (NGO), Helsinki, Finland; 5 Department of Research, Cancer Registry of Norway, Oslo, Norway

**Keywords:** Smoking, Cause-specific mortality, Population attributable fraction, NOWAC study, Norway, Women

## Abstract

Recent studies have found that the risk of death continues to increase among female smokers, as compared with women who have never smoked. We wanted to examine the effect of smoking on all-cause and cause-specific mortality and calculate the corresponding population attributable fraction (PAF) of mortality in the Norwegian women and cancer study; a nationally representative prospective cohort study. We followed 85,320 women, aged 31–70 years, who completed a questionnaire in 1991–1997, through linkages to national registries through December 2008. Questionnaire data included information on lifestyle factors, including lifetime history of smoking. Poisson regression models were fitted to estimate relative risks (RRs) with 95 % confidence intervals (CIs) adjusting for age, birth cohort, education, postmenopausal status, alcohol consumption and body mass index, all at enrollment. During a mean follow-up time of 14 years 2,842 deaths occurred. Compared with that of never smokers, current smokers had a mortality rate that was double (RR = 2.34; 95 % CI 2.13–2.62) from deaths overall, triple (RR = 3.30; 95 % CI 2.21–4.82) from cerebrovascular disease and myocardial infarction (RR = 3.65; 95 % CI 2.18–6.15), 12 times (RR = 12.16; 95 % CI 7.80–19.00) from lung cancer and seventeen times (RR = 17.00; 95 % CI 5.90–48.78) from chronic obstructive pulmonary diseases. The PAF of mortality due to smoking was 34 % (CI 30–39). In summary, one in three deaths among middle aged women in Norway could have been prevented if the women did not smoke. More middle-aged women, than ever before, are dying prematurely due to smoking in Norway.

## Introduction

Worldwide, tobacco use causes annually more than five million deaths, or one death every 6 seconds. Unless current smokers stop smoking before or during middle age, this number is expected to rise to eight million or more by the year 2030 [1–3]. In spite of the overwhelming evidence of the detrimental effects of smoking on health [4, 5], the tobacco use among women is rising globally and the age of initiation of daily smoking among women seems to have become as young as it is in men [6, 7].

For women in Norway, the prevalence of daily smokers was 23 % in 1954, the peak was at 37 % in 1970 and then it stabilized at around 32 % for the rest of the century [8]. The decline among female smokers first started in the twentyfirst century [9]. As a consequence the lung cancer incidence and mortality rates have both been increasing for Norwegian women. In fact, the strongest increase in cancer incidence from the past 5-year period (2001–2005) until the current one (2006–2010) occurred in lung cancer (16%) [10]. The four tobacco epidemic stages model suggested by Lopez et al. almost 20 years ago described the effects on mortality of the cigarette epidemic in Western countries such as the USA, UK and Australia. The rise and fall in female smoking and in smoking-attributed mortality usually lagged behind that in men by about 20–30 years [11].

Recent studies have found that for women the risk of death from cigarette smoking continues to increase resulting in a population attributable fraction (PAF) of mortality that is larger than previously reported [4, 12–15].

As pointed out by Oza et al. [16], estimating the number of deaths attributable to smoking is needed for priority setting and monitoring the disease burden associated with this risk factor in different populations.

Today, the majority of middle-aged Norwegian women are either former or current smokers, who started to smoke in their teens [17]. We wanted to estimate the effects of cigarette smoking on all-cause and cause-specific mortality and estimate the proportion of deaths attributable to smoking in the Norwegian Women and Cancer (NOWAC) study, a nationally representative prospective cohort study.

## Materials and methods

### Study population

The NOWAC study is a nationally representative prospective cohort study comprising a sample of the Norwegian middle-aged female population. The cohort profile has been previously described in detail [18, 19]. Briefly, a random sample of women was selected from the central population register according to year of birth. These women were sent a letter of invitation to participate in the study, which also contained a questionnaire, and a pre-stamped return envelope. The National Data Inspectorate and the Regional Committee for Medical Research Ethics approved the study. All women gave an informed consent (http://site.uit.no/kvinnerogkreft).

The inclusion criteria for the present study were women born from 1926 to 1965, who during 1991–1997 completed an initial questionnaire, containing questions on life style factors as smoking and alcohol consumption (N = 95,947). The overall response rate was 57 %. Women who emigrated or died (N = 22) before the start of follow-up were excluded. We excluded 10,605 women due to missing information on either smoking status (N = 2,224) or any of the co-variables (education, BMI, menopausal status and alcohol consumption) under study. Altogether 458 women with missing information had died during follow-up of which 64 (14 %) had missing information on smoking status. The analytical cohort comprised the remaining 85,320 women with complete information in the multivariable analyses. Compared with the women in the analytical cohort, the women that were excluded due to missing information were on average 2.7 years older, but were similar according to education, BMI and alcohol consumption (data not shown).

### Exposure information

The baseline questionnaire included a detailed assessment of lifetime smoking history. The questionnaires asked if the women had ever smoked, and those answering “yes” were asked the number of cigarettes smoked daily at different ages. Subsequently, they were asked if they currently smoked on a daily basis. We categorized ever smokers according to current and former smoking status at enrollment. All women who were neither current nor former *daily* smokers were classified as never smokers. The baseline questionnaires also asked about years of education, height and current weight (allowing us to calculate body mass index, BMI, kg/m^2^), menopausal status (pre-, peri-, postmenopausal), postmenopausal hormone therapy (yes, no), hysterectomy (yes, no), including alcohol consumption, all at enrollment. We calculated average consumption of alcohol in g/day based on the content of pure alcohol in different sorts of beverages and usual portion sizes in Norway among drinkers. Women who reported to be teetotalers and those answering “seldom” or “never” had their alcohol consumption set to zero.

### Follow-up and endpoints

We followed the women through linkages to the Norwegian Central Population Register, utilizing the unique national birth number to identify all cases of deaths and emigrations. The end point in our study was death from any cause during follow-up, which ended December 31st, 2008. All causes of death were coded according to the original codes in the International Classification of Diseases, Ninth Revision (ICD-9) before 1996 and the original codes of the ICD-10 from 1996 and after. The deaths were classified into three main outcome categories: death from cancer (C00–C97), cardio-vascular diseases (CVDs) (I00–I99), respiratory diseases (J00–J99). The other deaths were collapsed in one group.

Cancer deaths were further classified into: (1) lung cancer (C34), (2) other smoking-related cancers [5], which included oral cavity (C00–C08), oropharynx (C09, C10, C12–C14), nasopharynx (C11), esophagus (C15), stomach (C16), colorectal (C18–C21), liver (C22), pancreas (C25), nasal cavity and sinuses (C30, C31), larynx (C32), uterine cervix (C53), kidney (C64), lower urinary tract (C65–C68) and myeloid leukemia (C42) and (3) remaining cancers not established to be smoking-related [5]. Deaths from CVDs and respiratory diseases were further classified into: (1) myocardial infarction (MI), (I21–I22), (2) cerebrovascular diseases (I60–I69), (3) remaining circulatory diseases and (1) COPD (J40–J44), (2) remaining respiratory diseases, respectively.

### Statistical analysis

We used Poisson regression models (with attained age between cohort entry and exit as the underlying time variable) to obtain adjusted relative risks (RRs) with two-sided 95 % Wald confidence intervals (CI) that compared categories of smokers (current, former, ever) with never smokers treated as a fixed reference group. We fitted regression models by splitting the follow-up-time into 1-year intervals [20]. Risk time was counted from date of enrolment until censoring due to death, emigration or end of follow-up (December 31st, 2008), whichever occurred first. We estimated the age- and multivariable adjusted association between smoking status at enrolment and mortality overall and for 11 selected categories of disease outcome.

We included the following variables; years of education (<10,10–12, ≥13), BMI (<18.5, 18.5–24.9, 25–29.9, ≥30 kg/m^2^), menopausal status (pre-, peri-, postmenopausal, postmenopausal hormone therapy, hysterectomy), alcohol consumption (0, >0–4, ≥5 g/day) all at enrolment, and birth cohort (1927–1936,1937–1946,1947–1956, 1957–1965), decided a priori, for potential confounding the smoking and mortality association.

To indicate what proportion of the deaths in the population that would not occur if smoking were eliminated we calculated the PAF as described in the WHO global report [4], using the formula:

$${\text{PAF}} = \frac{{P_{\text{e}} (RR_{\text{e}} - 1)}}{{P_{\text{e}} \times RR_{\text{e}} + (1 - P_{\text{e}} )}}$$

where the notation *P*_e_ = the proportion of persons in the population exposed to the risk factor i.e. ever smokers and *RR*_e_ = the RR in the exposed compared to the unexposed group; i.e. ever compared with never smokers. We calculated the two-sided 95 % CI’s for the PAF’s using the delta method [21]. All statistical tests were two-sided and were considered statistically significant at *p* ≤ 0.05. The SAS© software version 9.22 was used for all statistical analyses.

## Results

Altogether 2,842 deaths occurred during the approximately 1.2 million woman-years of observation with a mean follow-up time of 14 years. Among the deaths, 1,800 (63 %) were due to cancer, 426 (15 %) due to CVD, 108 (4 %) due to respiratory diseases and 508 (18 %) to remaining causes. Overall, 65 % (n = 55,227) of the women reported to be ever (35 % current and 30 % former) smokers.

Table 1 shows the distribution of selected characteristics according to smoking status at enrollment. Current smokers were younger, had fewer years of education, a lower BMI and consumed more alcohol than never smokers (Table 1).

**Table 1:** Distribution of selected characteristics given as mean (SD)^a^ and percentages (%) according to smoking status, all at enrollment, Norwegian Women and Cancer (NOWAC) Study 1991–2008, (N = 85,320)

Characteristics	Smoking status		
	Never	Former	Current
Age at enrollment (years)^a^	46 (9.2)	46 (8.7)	44 (7.9)
Age at cohort exit (years)^a^	61 (8.4)	60 (8.0)	58 (7.7)
Person years of follow-up^a^	14 (4.0)	14 (4.1)	14 (4.4)
Education (years)^a^	12 (3.6)	12 (3.4)	11 (3.1)
BMI at enrollment^a^	24 (3.7)	24 (3.7)	23 (3.6)
Teetotallers (%)	40	23	22
Alcohol consumption (g/day)^b^	2 (3.9)	3 (5.8)	4 (7.0)

*SD* standard deviation, *BMI* body mass index

^a^Given as mean (SD)

^b^Among drinkers

Compared with never smokers, former smokers had a 34 % (RR = 1.34; 95 % CI 1.21–1.49) and current smokers a more than double (RR = 2.34; 95 % CI 2.13–2.62) overall mortality rate.

Both former and current smokers had, compared with never smokers, a significantly increased mortality rate from all three main outcome categories; cancer-, cardiovascular—and respiratory diseases. The association between smoking and the different mortality outcomes displayed in Table 2 was stronger for current than for former smokers. The highest increased mortality rate was from COPD (RR = 17.00; 95 % CI 5.90–48.78) when current was compared with never smokers (Table 2).

**Table 2:** Age- and multivariable^a^ adjusted overall and selected cause-specific relative risk (RR) of mortality according to smoking status at enrollment, Norwegian Women and Cancer (NOWAC) Study 1991–2008 (N = 85,320)

	Smoking status				
	Never	Former		Current	
	n = 30,093	n = 25,309		n = 29,918	
Mortality (n)	RR	RR	95 % CI	RR	95 % CI
*Overall mortality*					
Cases (N = 2842)					
Age adjusted	1.00	1.31	1.18–1.45	2.35	2.15–2.57
Multivariable^a^	1.00	1.34	1.21–1.49	2.34	2.13–2.62
*Three main outcome categories*					
Total cancer					
Cases (N = 1800)					
Age adjusted	1.00	1.15	1.02–1.30	1.91	1.70–2.13
Multivariable^a^	1.00	1.17	1.03–1.33	1.89	1.68–2.13
Total cardiovascular diseases					
Cases (N = 426)					
Age adjusted	1.00	1.57	1.19–2.06	3.67	2.87–4.68
Multivariable^a^	1.00	1.35	1.22–1.29	4.07	3.15–5.26
Total respiratory diseases					
Cases (N = 108)					
Age adjusted	1.00	3.47	1.72–6.99	10.78	5.68–20.45
Multivariable^a^	1.00	3.55	1.74–7.27	9.22	4.72–18.00
*Selected cause-specific categories*					
Lung cancer					
Cases (N = 298)					
Age adjusted	1.00	2.82	1.72–4.63	4.89	3.59–6.67
Multivariable^a^	1.00	2.40	1.43–4.03	12.16	7.80–19.01
Other smoking-related cancers^b^					
Cases (N = 764)					
Age adjusted	1.00	1.22	1.01–1.47	1.82	1.53–2.16
Multivariable^a^	1.00	1.24	1.03–1.51	1.81	1.50–2.17
Remaining^c^ cancers					
Cases (N = 738)					
Age adjusted	1.00	0.96	0.80–1.14	1.01	0.84–1.22
Multivariable^a^	1.00	1.07	0.90–1.27	1.09	0.90–1.31
Circulatory diseases					
Cases (N = 141)					
Age adjusted	1.00	2.10	1.24–3.55	5.62	3.52–8.96
Multivariable^a^	1.00	2.07	1.21–3.55	5.92	3.62–9.68
Myocardial infarction					
Cases (N = 103)					
Age adjusted	1.00	1.25	0.68–2.27	3.61	2.20–5.92
Multivariable^a^	1.00	1.11	0.59–2.09	3.65	2.18–6.15
Cerebrovascular disease					
Cases (N = 182)					
Age adjusted	1.00	1.47	1.00–2.17	2.74	1.92–3.92
Multivariable^a^	1.00	1.40	0.93–2.11	3.30	2.21–4.82
Chronic obstructive pulmonary diseases					
Cases (N = 68)					
Age adjusted	1.00	5.40	1.79–16.27	22.00	7.91–61.22
Multivariable^a^	1.00	5.02	1.63–15.48	17.00	5.90–48.78
Other respiratory diseases					
Cases (N = 40)					
Age adjusted	1.00	2.33	0.92–5.93	4.56	1.92–10.82
Multivariable^a^	1.00	2.71	1.05–6.98	4.51	1.78–11.42

*BMI* body mass index, *IARC* International Agency for Research on Cancer

^a^Adjusted for education (<10,10–12, ≥13 years), BMI (<18.5,18.5–24.9, 25–29.9, ≥30 kg/m^2^), menopausal status (pre-, peri-, postmenopausal, postmenopausal hormone therapy, hysterectomy), alcohol consumption (0, >0–4, ≥5 g/day), all at enrollment and birth cohort (1927–1936,1937–1946, 1947–1956, 1957–1964)

^b^Smoking-related cancers according to IARC 2012; i.e. oral cavity (C00–C08), oropharynx (C09, C10, C12–C14), nasopharynx (C11), esophagus (C15), stomach (C16), colorectal (C18–C21), liver (C22), pancreas (C25), nasal cavity and sinuses (C300, C31), larynx (C32), uterine cervix (C53), kidney (C64), lower urinary tract (C65–C68) and myeloid leukemia (C42), excluding lung cancer (C34)

^c^Remaining cancers not established to be smoking-related according to IARC 2012

Table 3 shows that the PAF of deaths among ever smokers was 34 % (95 % CI 30–39) compared with never smokers. The corresponding figure for lung cancer was 79 % (95 % CI 72–86) and for COPD 85 % (95 % CI 73–97) (Table 3).

**Table 3:** Overall and selected cause-specific mortality^a^ among ever compared with never smokers at enrollment, and the population attributable fraction (PAF) (%) of smoking with confidence intervals (CI), Norwegian Women and Cancer (NOWAC) Study, 1991–2008 (N = 85,320)

	Smoking status		Population	
	Never	Ever	PAF	
	n = 30,093	n = 55,227	%	CI
*All-cause mortality*			34	30–39
Relative risk	1.00	1.81		
		1.66–1.98		
*Three main outcome categories*				
Total cancer			24	18–31
Relative risk	1.00	1.50		
		1.35–1.67		
Total cardiovascular disease			50	40–59
Relative risk	1.00	2.52		
		1.99–3.20		
Total respiratory diseases			76	63–88
Relative risk	1.00	5.78		
		3.04–10.99		
*Selected cause-specific categories*				
Lung cancer			79	72–86
Relative risk	1.00	6.87		
		4.46–10.60		
Smoking-related cancers^b^			24	14–34
Relative risk	1.00	1.49		
		1.27–1.76		
Remaining^c^ cancers			3	0.9–15
Relative risk	1.00	1.05		
		0.89–1.23		
Circulatory diseases			63	49–77
Relative risk	1.00	3.59		
		2.26–5.69		
Myocardial infarction			43	21–65
Relative risk	1.00	2.17		
		1.33–3.54		
Cerebrovascular diseases			42	25–58
Relative risk	1.00	2.10		
		1.49–2.95		
Chronic obstructive pulmonary diseases			85	73–97
Relative risk	1.00	9.87		
		3.53–27.64		
Other respiratory diseases			61	34–88
Relative risk	1.00	3.41		
		1.45–7.99		

*BMI* body mass index, *IARC* International Agency for Research on Cancer

^a^Adjusted for education (<10,10–12, ≥13 years), BMI (<18.5,18.5–24.9, 25–29.9, ≥30 kg/m^2^), menopausal status (pre-, peri-, postmenopausal, postmenopausal hormone therapy, hysterectomy), alcohol consumption (0, >0–4, ≥5 g/day), all at enrollment and birth cohort (1927–1936,1937–1946, 1947–1956, 1957–1964)

^b^Smoking-related cancers according to IARC 2012; i.e. oral cavity (C00–C08), oropharynx (C09, C10, C12–C14), nasopharynx (C11), esophagus (C15), stomach (C16), colorectal (C18–C21), liver (C22), pancreas (C25), nasal cavity and sinuses (C300, C31), larynx (C32), uterine cervix (C53), kidney (C64), lower urinary tract (C65-C68) and myeloid leukemia (C42), excluding lung cancer (C34)

^c^Remaining cancers not established to be smoking-related according to IARC, 2012

## Discussion

Our study shows that compared with never smokers, both former and current smokers at cohort enrollment in the 1990s have an increased risk of dying overall, as well as dying from the three main outcome categories; total cancer, total CVD and total respiratory diseases. The increased risk was more pronounced for current than for former smokers indicating the effects of quitting smoking. One in three deaths in this female population may be attributed to smoking and could have been avoided if the women did not smoke.

As we do, recent studies [13–15, 22–27], including Caucasian women born in the 1940’s or later, found a more than doubling in the all-cause mortality rate, when comparing current with never smokers. Corresponding figures have for the first time emerged among Japanese women [28].

In our study, except for cancers not established to be smoking-related [5], current smoking was associated with an significantly increased risk of mortality from all categories of cause-specific mortality examined (lung cancer, other smoking-related cancers, circulatory diseases, MI, cerebrovascular diseases, COBP, and other respiratory diseases).

We find a three-fold mortality rate among current smokers compared with that of never smokers for cerebrovascular diseases and MI, similar to those displayed in the Nurses’ Health study [25]; and in the Million Women Study [13], while our mortality rate from COPD and from lung cancer was much lower than in the other two studies. The US study [25], had more than 12,000 deaths during a follow-up time of 24 years, while the UK study [13], had 66,000 deaths after 12 years. Both studies [25] had a RR of mortality from lung cancer of more than 20, while the mortality rate from COPD was 40 and 35, respectively. We expect that the deaths related to lung cancer and COPD, which have a long natural history, will increase when we have a longer follow-up time. Also, Kenfield et al. [26] showed in a later follow-up study that exposure assessed only at enrollment, underestimated the mortality risk due to smoking, compared with a second assessment during follow-up, especially for COPD and lung cancer.

The PAF of mortality due to smoking among Norwegian women has previously been estimated to be less than 20 % when mortality rates from lung cancer were used as an indirect measure of cigarette smoking [4, 12, 29], when cohort studies from the mid-70 s were used [23] and when the smoking prevalence surveys published by the Norwegian Central Bureau of Statistics were used [8, 30] Thun et al. [12], estimated that in 2009, the PAF of mortality due to smoking was highest (26–30 %) among women aged 35–69 years, in The Netherlands, Denmark, Hungary and Canada. In our study, based on individual data on both exposure and outcome, the corresponding PAF was estimated to be 34 % in Norway in 2008, with the upper limit of the 95 % CI as high as 39 %.

The life expectancy for Norwegian females was more than 83 years in 2008 [31]. The low background mortality rate for women, which is the denominator of our RRs, leads to greater PAF’s of mortality due to smoking. Our finding of a high proportion of deaths that could have been avoided if nobody was smoking is in accordance with the rapidly increasing lung cancer incidence for Norwegian women. The age adjusted world lung cancer incidence rate more than doubled from 11.7 per 100,000 person-years in the 5-year period (1986–1990) immediately before the commencement of our study to 25.1 per 100,000 person-years for the current 5 year period including the year which our follow-up ended [10]. This increasing lung cancer incidence also tells us that the PAF of mortality due to smoking among Norwegian women has not peaked yet.

As pointed out by Jha [3], the full effects of smoking can take 50 years to measure in individuals, and up to 100 years to measure in populations. Our study shows the PAF due to smoking among females already to be higher than the peak in the gender specific model recently developed by Thun et al. [12].

### Strengths

The most important strength of our study is that it is a nationally representative prospective cohort study allowing us to calculate the PAF of mortality due to smoking for middle-aged women. We know from our previous studies that the smoking exposure [17, 32, 33] and the cancer incidence [19] reflect known smoking patterns [8, 9] and cancer incidence [10] for Norwegian women. Thus, we are confident that our cohort is representative of the Norwegian female population, born between 1926 and 1965, both according to exposure and outcome of this study.

Other major strengths of our study are; we have a high proportion of both current and former smokers, virtually complete follow-up through the National population based registries and the possibility of examining the association with smoking according to both all-cause and cause-specific mortality. Another force is that we focus our PAF estimates on the comparison between ever versus never smokers. Thus, it is only never smokers that could possibly change smoking status during follow-up. Since very few Norwegians start to smoke after the age of 30 and the mean age at enrolment for our study is more than 40 years, we are confident that the possible changes in smoking status among the never smokers during follow-up did not influence our PAF estimates. Furthermore, we have detailed information on, and were able to control for alcohol consumption and BMI, which are established risk factors for mortality.

### Limitations

One major limitation of this report is that we have a limited number of deaths. Furthermore, 9 % of the women had missing values for physical activity levels and therefore this variable was not included in the multivariable analyses as there was no difference in physical activity levels according to smoking status. As in our study, neither of the seven [13–15, 25, 26, 34, 35] recently published studies did find much effect of confounding when they compared the effect of smoking on mortality. Five of these studies were conducted in the US [14, 15, 25, 26, 34]; one in the UK [13], and one in China [35]. We found a small non-significantly increased risk of deaths from cancers not established to be associated with smoking. This may be due to chance or that breast cancer should be in the category for smoking-related cancers. According to the most recent IARC monograph cigarette smoking is possibly carcinogenic to the human breast [5]. After this monograph was published two large cohort studies, one from the US [36], and our own Norwegian study [37] based on more than 300,000 women, conclude that smoking initiation before the first childbirth increase the risk of breast cancer.

Other limitations are that we have not validated self-reported information on height and body weight and that the current PAF formula does not provide a fully adjusted estimate of PAF due to lack of adjustment of the prevalence. We chose not to exclude women with prevalent disease as a previous study from the same cohort which examined physical activity and mortality found basically the same results when they did their analyses with and without women with prevalent disease at enrolment [38]. This has most likely deflated or RR estimates of mortality. Given the reduction in life expectancy associated with smoking we cannot rule out that part of the cause-specific associations is hidden or obscured by the competing causes of death with increasing age. We do not expect that the changes in BMI or alcohol consumption during follow-up would influence our results to any great extent. Nevertheless, we cannot rule out the possibility of residual confounding due to the above described factors, or other factors we did not measure.

In summary, one in three deaths among middle aged women in Norway could have been prevented if the women did not smoke. More middle-aged women, than ever before, are dying prematurely due to smoking in Norway.

## Abbreviations

PAF: Population attributable fraction
RR: Relative risk
CI: Confidence interval
NOWAC study: Norwegian women and cancer study
IARC: International Agency for Research on Cancer
BMI: Body mass index
CVD: Cardio-vascular diseases
MI: Myocardial infarction
COPD: Chronic obstructive pulmonary disease
ICD: International classification of diseases
SD: Standard deviation
